# Pharmacogenetics of FSH Action in the Female

**DOI:** 10.3389/fendo.2019.00398

**Published:** 2019-06-26

**Authors:** Alessandro Conforti, Alberto Vaiarelli, Danilo Cimadomo, Francesca Bagnulo, Stefania Peluso, Luigi Carbone, Francesca Di Rella, Giuseppe De Placido, Filippo Maria Ubaldi, Ilpo Huhtaniemi, Carlo Alviggi

**Affiliations:** ^1^Department of Neuroscience, Reproductive Science and Odontostomatology, University of Naples Federico II, Naples, Italy; ^2^G.E.N.E.R.A. Centre for Reproductive Medicine, Clinica Valle Giulia, Rome, Italy; ^3^Medical Oncology, Department of Senology, National Cancer Institute, IRCCS Fondazione G. Pascale, Naples, Italy; ^4^Department of Surgery and Cancer, Institute of Reproductive and Developmental Biology, Imperial College London, London, United Kingdom; ^5^Istituto per l'Endocrinologia e l'Oncologia Sperimentale (IEOS) Consiglio Nazionale delle Ricerche, Naples, Italy

**Keywords:** FSH, FSH receptor, polymorphisms, mutations, ovarian stimulation, assisted reproductive technology, IVF, genetic variants

## Abstract

The purpose of a pharmacogenomic approach is to tailor treatment on the basis of an individual human genotype. This strategy is becoming increasingly common in medicine, and important results have been obtained in oncologic and antimicrobial therapies. The rapid technological developments and availability of innovative methodologies have revealed the existence of numerous genotypes that can influence the action of medications and give rise to the idea that a true “individualized” approach could become in the future a reality in clinical practice. Moreover, compared to the past, genotype analyses are now more easily available at accessible cost. Concerning human reproduction, there is ample evidence that several variants of gonadotropins and their receptors influence female reproductive health and ovarian response to exogenous gonadotropins. In more detail, variants in genes of *follicle-stimulating hormone* β*-chain* (FSH-B) and its *receptor* (FSH-R) seem to be the most promising candidates for a pharmacogenomic approach to controlled ovarian stimulation in assisted reproductive technologies. In the present review, we summarize the evidence regarding FSH-B and FSH-R variants, with special reference to their impact on reproductive health and assisted reproductive technology treatments.

## Introduction

Follicle-stimulating hormone (FSH) is a pituitary gonadotropic hormone, which is fundamental for follicle growth in females and spermatogenesis in males. FSH is an heterodimeric molecule belonging to the glycoprotein hormone family. It consists of the common α-subunit shares as with other glycoprotein hormones (LH, hCG, TSH) and the hormone specific β subunit. FSH, when in the ovary and testis, binds to its cognate receptor (FSH-R), which belongs to the superfamily of the G-protein coupled receptors. It is characterized by a long ligand-binding extracellular domain, seven transmembrane domains, mediating the hormonal stimulus, and an intracellular C-terminal domain participating in receptor internalization and desensitization of the signal. Through the interaction with its receptor, FSH activated several intracellular signaling pathways, the most important of them being adenylyl cyclase and β-arrestins (1, 2).

It has recently been demonstrated that FSH exerts its action outside the reproductive tract, including in the placenta, hepatocytes and tumor blood vessels (3, 4). In addition, FSH was demonstrated to be involved in the pathogenesis of endometriotic lesions (5). Focusing on the female reproductive tract, it was also recently demonstrated that FSH could exert its effect on the endometrial glands. In detail, FSH-R was able to increase in these cells intracellular levels of cAMP, leading to induction of steroidogenesis (6). During the menstrual cycle, FSH has several important actions. Firstly, it promotes folliculogenesis by stimulating estradiol production by the aromatase enzyme system, stimulating granulosa cell growth and inducing the expression of luteinizing hormone receptors (1). Together with LH, FSH levels peak in the mid-cycle which induces important actions in the ovulation process, such as the stimulation of proteolytic enzymes essential for follicular wall rupture (7). Finally, in the early follicular phase FSH is involved in the recruitment of new antral follicles for the next cycle of folliculogenesis (8). On the basis of differences in the terminal sialic acid residues in the carbohydrate moieties that are attached to the FSH protein, numerous isoforms of FSH have been identified (9). Acidic isoforms seems to be involved in follicular recruitment at the end of menstrual cycles, while follicle selection and rupture seem to be promoted by basic FSH isoforms (9). Given its biological importance in folliculogenesis, pharmaceutical FSH products are currently adapted for multiple follicular growth in assisted reproductive technology (ART) (10). The ovarian activity, as well as the ovarian response to exogenous gonadotropin appear to be influenced by specific genetic traits involving gonadotropins and their receptors (11–14) (Table 1). In the present review, we will summarize the most important evidence concerning variants of FSH and FSH-R and their implication in female reproductive functions.

**Table 1:** Clinical manifestations and pathogenetic effects of FSH-R and FSH-B subunit most common variants (https://www.ncbi.nlm.nih.gov/snp).

**Polymorphisms (reference SNP)**	**RefSeqGene**	**Locus reference genomic sequence (LRG)**	**Variant**	**Pathogenetic effect**	**Clinical manifestations**	**References**
*FSH-R RS1394205 (likely pathogenic)*	NG_008146.1:g.5046 G>A	LRG_536	A	Reduced transcription activity; Reduced protein levels	Higher consumption of gonadotropin during controlled ovarian stimulation; Reduced number of oocytes retrieved	(15) (16) (3) (17)
*FSH-R RS6166 (likely pathogenic)*	NG_008146.1:g.196710 G>A	LRG_536	G	Impaired ligand sensitivity	Reduced number of oocytes retrieved Higher consumption of gonadotropin during controlled ovarian stimulation	(15) (18) (19) (20) (21)
*FSH-R RS6165 (likely pathogenic)*	NG_008146.1:g.195590 G>A	LRG_536	G	Impaired ligand sensitivity	Reduced number of oocytes retrieved Higher consumption of gonadotropin during controlled ovarian stimulation Lower number of embryos	(15) (22) (23)
*FSH-B RS6169 (uncertain significance)*	NG_008144.1:g.7623 C>T	Not available	C	Unknown	Polycystic ovarian syndrome development No effect detected during controlled ovarian stimulation	(24) (13) (25)
*FSH-B RS10835638 (likely pathogenic)*	NG_008144.1:g.4790 G>T	Not available	T	Decreased trascriptional activity	Reduced FSH basal levels Longer menstrual cycles Delayed menopause	(26) (27) (28)

## Methods

A systematic search was carried out using MEDLINE (Pubmed) AND Scopus databases with no restriction of language or time period. The search strategy consisted in the use of the combinations of the following keywords: “controlled ovarian stimulation,” “ART,” “IVF,” “ICSI,” “FIVET,” “IUI,” “intrauterine insemination,” “ovulation induction,” “ovarian stimulation,” “polymorphism,” “SNV,” “Single nucleotide variant,” “FSH Receptor,” “FSHR,” “FSH,” “follicle-stimulating hormone,” “follicle-stimulating hormone,” and “beta subunit.”

In view of the recent meta-analysis published by our group (15) we updated our research adding also more recent papers in the present review (29–32).

As recommended by Human Genome Variation Society (33) every single nucleotide variants (SNV) illustrated in the present paper was reported indicating Locus Reference Genomic sequence (LRG) and RefSeqGene or transcript (Table 1).

### Genetic Variants of FSH Beta Subunit

In contrast to LH beta subunit, FSH-B appears to be highly conserved (34). Indeed, few variants of the gene encoding for FSH-B subunit have been identified so far. The first variant was identified in 1993 in a women with primary amenorrhea, sexual infantilism and infertility (35, 36). This variant consisted in a two-nucleotide deletion in codon 61 that gave rise to premature stop codon. Thereafter, several other inactivating variants have been identified. Most of them induce an alteration of the cysteine knot structure of FSH which is crucial for its biological activity (34). Thus, the majority of variants inactivating FSH-B are characterized by the absence of puberty, infertility and the absence of breast maturation and few of them show partial puberty development (34, 37).

Considering the conserved structure of FSH, very few clinically significant variants have been identified. Among the 24 SNVs identified (18) only the one located in FSH-B chain promoter (*C.-221G*>*T, RS10835638*) seems to have significant clinical impact on male and female reproduction (38, 39). In the first report by Grigorova et al. T homozygous men showed lower FSH levels and reduced testicular volume than other haplotypes (38). Conversely, in 365 women with normal menses the T homozygotes showed elevated FSH and LH levels with reduced progesterone production (39). In another study, the T allele resulted in longer menstrual cycles [0.16 Standard differences; 95% confidence interval (CI) 0.12–0.20; *P* < 0.05], in delayed age at menopause (0.13 years; 95% CI 0.04–0.22; *P* < 0.05), and greater female nulliparity [odds ratio (OR) = 1.06; 95% CI 1.02–1.11; *P* < 0.05] (26). Interestingly, the same study showed lower risk of endometriosis among T carriers compared with other haplotypes [OR = 0.79; 95% CI 0.69–0.90; *P* < 0.05]. In another study, involving 193 infertile eumenorrheic women, a statistically significant reduction of FSH on cycle day 3 was observed in carriers with the combination of FSH-B *(C-211 G*>*T, RS10835638)* GT + TT/FSH-R (*C.2039 G*>*A, RS6166*) AA genotype, compared with the FSH-B GG/FSH-R GG genotype (27). More recently, it was confirmed that the T allele carriers were associated with higher FSH and LH levels and idiopathic infertility (40). The T allele of FSH-B *C-211 G*>*T, RS10835638* appears to decrease transcriptional activity of the gene (28). Very recently, Trevisan et al. observed in a cross-sectional study involving 140 infertile women (median age 33 years), that women carrying GT genotype (*n* = 38) had a lower response to ovarian stimulation compared to GG (wild type) genotype (*n* = 102) with of number of oocytes retrieved (3.0 vs. 5.0, *p* = 0.03) and a lower number of embryos at the end of stimulation (2 vs. 3, *p* = 0.02) (29).

There is also evidence which suggests that another variant of FSH-B subunit *(C.228 C*>*T, RS6169)* might be implicated in the development polycystic ovarian syndrome (24, 25). A retrospective analysis of 135 Chinese women between 19 and 38 years of age affected by PCOS with 105 as a normal control, a higher prevalence of homozygous carrier was observed in PCOS than in the control group (12.6 vs. 3.8%) (25). Furthermore, the frequency of this variant was more pronounced in a specific subgroup of PCOS women, namely those with obesity (0.50 and 31.0%, respectively) and hyperandrogenism. These findings support the concept that hyperandrogenic PCOS women could show peculiar characteristics and probably display specific pathogenetic mechanisms (41). In a recent prospective trials involving 30 normogonadotropic women, we did not observe differences in terms of ovarian response or pregnancy rate when comparing different haplotypes of this variant (13). Despite the low number of patients recruited, our study did not support any implications in terms of ovarian response to exogenous gonadotropin and ART success.

### Genetic Variants of FSH Receptor

Several inactivating and activating variants of FSH-R have been identified (42). The majority of the inactivating variants are located on exons 7 and 10 (42). The most typical clinical manifestations are primary amenorrhea, elevated FSH levels, and infertility. Specific inactivating variants were also associated with polycystic ovarian syndrome (43). Also, the majority of activating variants are located in exon 10. The most common clinical manifestation is a spontaneous occurrence of ovarian hyperstimulation syndrome. In general, while activating variants in the FSH-R gene can manifest in heterozygotes, the inactivating variants alter the phenotype only when present in the homozygous or compound heterozygous form Desai et al. (42).

The FSH-R gene carries more than 2000 single nucleotide variants (SNVs) (18). Among them the most widely studied common variants which apparently impact on female reproduction are: -*29 G*>*A. (RS1394205); C.919G*>*A (RS6165); C.2039G*>*A (RS6166)*. Two FSH-R variants with SNVs in the coding region have been identified and well-characterized (44). The SNV known as the Serine680 variant causes the replacement of asparagine (Asn) for serine (Ser) at the 680 position, which is located in the intracellular domain of the FSH-R protein. The *RS6165* SNV replaces threonine (Thr) by alanine (Ala). Except in some African populations, the two SNVs are in linkage disequilibrium (19); this means that carriers who possess Thr307 nearly always have Asn680 present on the same allele and carriers who have Ala307 have Ser680 on the same allele (45). The former (*RS6166*) introduces a potential phosphorylation site and the latter (*RS6165*) results in a change from a polar to a non-polar hydrophobic amino acid, thereby removing a potential O-linked glycosylation site (19). *In vitro* studies conducted using human granulosa cells showed that GG carriers of the FSH-R (*RS6166*) genotype have greater resistance to FSH than do AA carriers (18, 46) and are characterized by slower kinetics of cAMP production, ERK1/2, and CREB phosphorylation (47). Despite the linkage disequilibrium between these two SNVs, several studies suggest that these two variants could influence ovarian stimulation (OS) outcome in different ways. In detail, Achrekar et al. observed that only the FSH-R *RS6165* variant could significantly impact the total FSH consumption during OS (3). Discrepancies between FSH-R (*RS6166*) and FSH-R (*RS6166)* were also reported by Trevisan et al. in a cross-sectional study involving 149 infertile women, in which a difference in terms of the number of embryos produced was observed only among different FSH-R (*RS6165*) haplotype (23).

Our findings in a recent systematic review corroborate these previous observations. Indeed, we found that GG FSH-R (*RS6166*) carriers had higher ovarian resistance to exogenous gonadotropin and, consequently, had fewer oocytes compared with AA carriers (15). These findings were also confirmed in a more recent study (31). In addition, higher FSH basal levels and resistance to clomiphene citrate were observed in G allele carriers, supporting an higher receptorial resistance even to endogenous level of FSH (15, 27, 48, 49). Conversely, A allele carriers show an higher FSH sensitivity as confirmed in a recent retrospective study of 586 infertile women undergoing their first IVF cycle, where an increased risk for developing OHSS syndrome (OR 1.7 95% CI 1.025–2.839, *p* = 0.04) was observed in carriers of this allele (30).

The finding of Borgbo et al. that the FSH-R *RS6166* and FSH-R *RS6165* GG carriers had higher *LHCGR* gene expression but lower *Anti-Müllerian hormone receptor-2* expression vs. carriers of the other haplotypes, suggested that these variants could affect the protein expression of human antral follicles (50). Nonetheless, it remains to be established whether FSH-R *RS6166* and *RS6165* influence expression of the FSH-R protein.

The FSH-R−29 G>A (RS1394205) variant is located in the 5′-untranslated region of the gene and is able to influence ovarian response. *In vitro* studies showed that A allele presence is characterized by reduced mRNA transcriptional activity and reduced FSH-R protein level (46, 47).

In ART context, Achrekar et al. reported with homozygous variant genotype AA lower number of oocytes and lower pregnancy rate compared with GG genotype in women who underwent OS (22). This observation was confirmed in a further larger study by Desai et al. involving 100 women (16) where those with the AA genotype at position−29 were at higher risk for poor ovarian response in comparison to the other haplotypes (OR 8.63, 95% CI 1.84–45.79; *P* = 0.001). In contrast, other authors did not confirm significant effects of this variant concerning the ovarian response (31, 32, 51). The evidence regarding the clinical effect of the *FSH-R*−*29 G*>*A* (*RS1394205*) variant on OS was summarized in a recent meta-analysis, which showed that higher exogenous FSH consumption is required in homozygotes for the A allele than carriers of the G allele (15).

### Is There Need for a Pharmacogenomic Approach in ART?

Demographic and anthropometric characteristics of women and the ovarian reserve tests do not fully explain the ovarian response to exogenous gonadotropin (52, 53). Indeed, there is a subgroup of women that, despite showing normal ovarian reserve in terms of functional and biochemical markers, have an “unexpected” impaired prognosis to ART and poor or suboptimal number of oocytes at the end of stimulation (54). This ovarian resistance to exogenous gonadotropin is also called “hypo-response,” and it was recently included in the new POSEIDON classification of low prognosis patients in ART (55–58).

**Figure 1:**
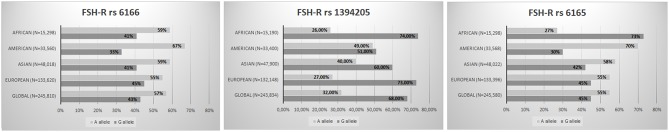
FSH-R variants worldwide distribution (*RS6166; RS1394205; RS6165*) (The Genome Aggregation Database—https://www.ncbi.nlm.nih.gov/snp/).

**Figure 2:**
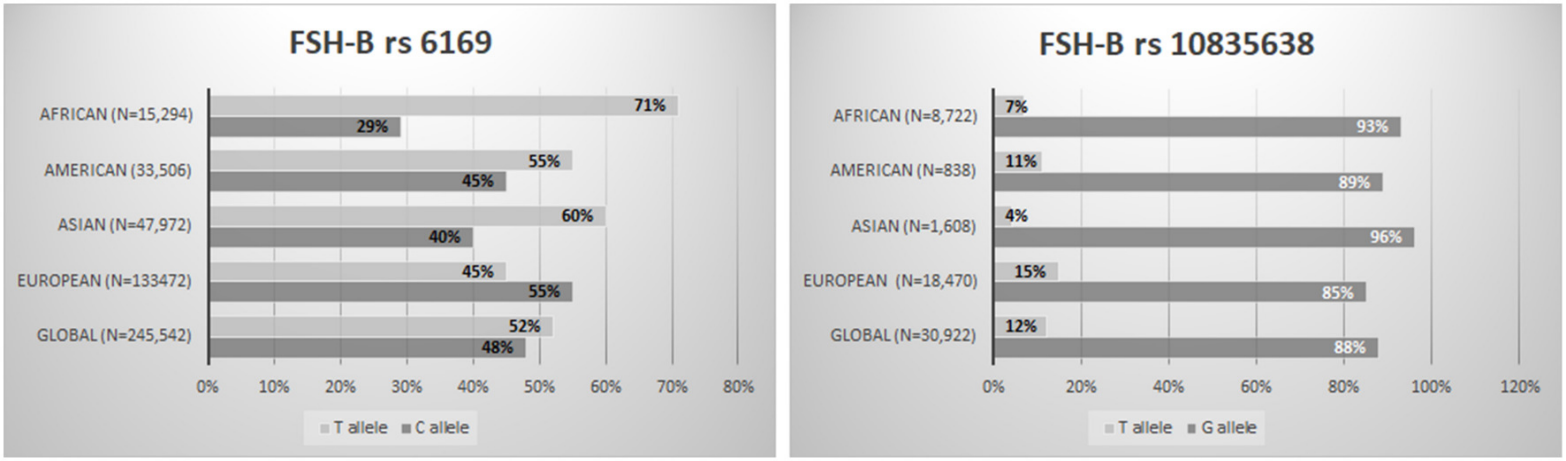
FSH-B subunit variants worldwide distribution (*RS6169; RS10835638*) (The Genome Aggregation Database—https://www.ncbi.nlm.nih.gov/snp/).

The fact that several variants could in some way affect the ovarian response to OS opens up the way to a pharmacogenomic approach to ART and might partially explain the hypo-response phenomenon (13, 59). The pharmacogenomic approach is spreading more and more to several fields, and it could provide the explanations for adverse or poor drug effects (60). In the ART scenario, a pharmacogenomic approach to OS could optimize ART treatments, thereby reducing both poor response rates and potentially life-threatening excessive ovarian responses (53). However, although more than 30 studies have already been published, no large randomized clinical trials on this topic have been conducted, indicating that the pharmacogenomic approach to OS is still a largely neglected topic. In addition, it should be underlined that variants, especially those involving FSH receptors are very common in general population (19, 61–63) (Figures 1, 2). Furthermore, the genotype analysis can now be provided at low cost (15). So far, few studies have adopted a pharmacogenomic approach to ART with the FSH-R variant rs6166 being the one most often investigated (2, 20). The first one was conducted by Behre et al. (21). In detail, women undergoing controlled ovarian hyperstimulation for ART, homozygous for the wild-type or for the FSH-R SNV(*C.2039G*>*A [RS6166])*, were randomly assigned to group I (GG carriers, *n* = 24), receiving an FSH daily dose of 150 U/day, or group II (GG carries, *n* = 25), receiving an FSH dose of 225 U/day. Age- and body mass index-matched AA carriers, receiving a daily dose of 150 IU, served as control group. The wild-type group (AA carriers) had higher estradiol production after treatment with 150 IU/day of FSH compared with the GG carriers who received the same dose. The increment of dosage for 150–225 IU/day was able to compensate for this discrepancy. This finding was confirmed by those reported by Genro et al. (64). The authors showed that the follicle development during OS was not significantly influenced by the presence of *FSH-R RS6166/RS6165* when a high FSH dose (300 IU per day) was administrated during OS (64). These results suggested that increasing FSH dosage during OS could mitigate the negative effect exerted by the FSH-R variant on the ovarian response. Finally, a recent meta-analysis including 4,425 observation concluded that GG allele carriers produced a significantly lower number of oocytes compared with AA (Random Weight Mean Difference: 0.84, 95% CI: 0.19–1.49, *P* = 0.01, *P* = 0.03) and AG carriers (Random Weight Mean Difference: 0.88, 95% CI: 0.12–1.63, *P* = 0.02) (15). Furthermore, gonadotropin type seems to influence the number of oocytes collected in relation to the FSH-R (*RS6166*) genotype distribution. As a matter of fact, the number of oocytes retrieved was significantly higher in AA carriers than in GG carriers when recombinant FSH was used, but not when urinary FSH formulations with LH activity were used (15). Although this finding suggests that the addition of LH in OS (65–67) could also mitigate the effects of FSH-R *RS6166* variants more data are needed on this issue.

## Conclusion

In conclusion, increasing evidence indicates that a pharmacogenomic approach to ovarian stimulation could become a clinical reality in the future. So far, specific variants of FSH-B and FSH-R represent promising genetic markers to better standardize controlled ovarian stimulation in women undergoing ART.

## Author Contributions

AC and CA idealized the paper and wrote the first draft. All authors participated in literature research and paper editing. The senior authors IH and GD supervised the paper and participate in the elaboration of the final version. All authors listed have made intellectual contribution to the work and approved the final version.

### Conflict of Interest Statement

The authors declare that the research was conducted in the absence of any commercial or financial relationships that could be construed as a potential conflict of interest.
